# Glucose-6-phosphate dehydrogenase and transketolase modulate breast cancer cell metabolic reprogramming and correlate with poor patient outcome

**DOI:** 10.18632/oncotarget.21601

**Published:** 2017-10-07

**Authors:** Adrián Benito, Ibrahim H. Polat, Véronique Noé, Carlos J. Ciudad, Silvia Marin, Marta Cascante

**Affiliations:** ^1^ Department of Biochemistry and Molecular Biomedicine, Faculty of Biology, Universitat de Barcelona, Barcelona, Spain; ^2^ Institute of Biomedicine of Universitat de Barcelona (IBUB) and CSIC-Associated Unit, Barcelona, Spain; ^3^ Department of Biochemistry and Physiology, Faculty of Pharmacy and Food Sciences, University of Barcelona, Barcelona, Spain

**Keywords:** breast cancer, tumor metabolism, pentose phosphate pathway, transketolase, glucose-6-phosphate dehydrogenase

## Abstract

The pentose phosphate pathway is a fundamental metabolic pathway that provides cells with ribose and NADPH required for anabolic reactions — synthesis of nucleotides and fatty acids — and maintenance of intracellular redox homeostasis. It plays a key role in tumor metabolic reprogramming and has been reported to be deregulated in different types of tumors. Herein, we silenced the most important enzymes of this pathway — glucose-6-phosphate dehydrogenase (G6PD) and transketolase (TKT) — in the human breast cancer cell line MCF7. We demonstrated that inhibition of G6PD, the oxidative branch-controlling enzyme, reduced proliferation, cell survival and increased oxidative stress. At the metabolic level, silencing of both enzymes reduced ribose synthesis. G6PD silencing in particular, augmented the glycolytic flux, reduced lipid synthesis and increased glutamine uptake, whereas silencing of TKT reduced the glycolytic flux. Importantly, we showed using breast cancer patient datasets that expression of both enzymes is positively correlated and that high expression levels of G6PD and TKT are associated with decreased overall and relapse-free survival. Altogether, our results suggest that this metabolic pathway could be subjected to therapeutic intervention to treat breast tumors and warrant further investigation.

## INTRODUCTION

Cancer cells need to reprogram their metabolism to fulfill specific metabolic requirements and to achieve a fully malignant phenotype [1, 2]. The so-called tumor metabolic reprogramming is aimed to meet the bioenergetics demands of cancer cells, providing them with precursors for the synthesis of macromolecules and maintaining redox homeostasis [3]. One of the most important metabolic pathways that participate in these processes is the pentose phosphate pathway (PPP), which synthesizes the nucleotide precursor ribose-5-phosphate and produces the reduced form of the nicotinamide adenine dinucleotide phosphate (NADPH), an essential cofactor required for the synthesis of lipids and the maintenance of the antioxidant systems, such as the reduced glutathione pool. Thus, it has been proposed that the activation of the PPP could be regarded as a hallmark of cell transformation [4].

The pentose phosphate pathway is divided into the oxidative and the non-oxidative branches. The oxidative branch (ox-PPP) catalyzes the irreversible transformation of glucose-6-phosphate (G6P) into ribulose-5-phosphate (Ri5P) with the subsequent production of NADPH and CO_2_. The non-oxidative branch (nonox-PPP) is a reversible pathway that interconverts glyceradehyde-3-phosphate (G3P), erythrose-4-phosphate (E4P), xylulose-5-phosphate (X5P), ribose-5-phosphate (R5P), fructose-6-phosphate (F6P) and sedoheptulose-7-phosphate (S7P), contributing to the synthesis of Ri5P and the redirection of the excess of this metabolite towards glycolysis. Glucose-6-phosphate dehydrogenase (G6PD) and transketolase (TKT) are considered as the key enzymes of the oxidative and the non-oxidative branches, respectively.

G6PD is a fundamental enzyme for protecting cells against oxidative stress [5–8] and it is involved in some biosynthetic processes, such as lipogenesis, by providing NADPH [9, 10]. Its expression and activity have been reported to be regulated by some of the most important oncogenes such as *k-ras* [11] or *p53* [2, 12] and it is one of the core enzymes involved in the response to oxidative stress coordinated by NRF2, a critical transcription factor reported to play a key role in tumorigenesis [9, 13, 14]. Compared to normal cells, malignant cells display higher levels of endogenous oxidative stress both *in vitro* and *in vivo* [15–17]. Particularly, breast tumors are characterized by persistent ROS generation [18, 19] and markers of constitutive oxidative stress have been detected in patient samples of breast carcinomas [20–22]. Consequently, breast tumors also display greater dependence on ROS detoxification systems, which increases gradually as the tumor progresses and becomes metastatic [23]. Under high oxidative stress conditions, G6PD activity increases to produce NADPH needed for the generation of reduced glutathione. Under these conditions, TKT converts the excess of R5P to G3P and F6P by a series of reactions. In this way, F6P can then be converted to G6P to replenish the ox-PPP for additional NADPH generation while G3P can be metabolized through further steps of glycolysis [10]. In cancer cells, given their rapidly dividing nature, TKT activity is increased to produce additional R5P from F6P and G3P by reverse reactions. In fact, it has been demonstrated that in rapidly growing cancer cells, 80% of ribonucleotides are produced through the nonox-PPP [24]. TKT activity has been reported to have a high control coefficient of tumor growth in mice with Ehrlich’s ascites tumor [25] and to be increased in (pre)neoplasic lesions in rat liver [26] and pancreatic adenocarcinoma, where the nonox-PPP is the main pathway responsible for riboneogenesis [24, 27]. Similar to G6PD, TKT is also overexpressed with NRF2 activation to assure sufficient NADPH and nucleotide levels. Well-known oncogenes such as K-Ras, B-Raf, Myc and PI3K/Akt are indirect regulators of TKT, since they take role in expression of NRF2 [9, 13]. Altogether, both G6PD and TKT are crucial enzymes in modulating the tumor metabolic phenotype.

The purpose of this study is to investigate the function of these two enzymes in breast cancer cell metabolism and to explore their potential as therapeutic targets. In our study, we chose breast cancer cells because they heavily rely upon PPP to manage oxidative stress and survive [10, 28]. Particularly, we selected MCF7 cells because they reportedly show increased expression of G6PD compared with the near-normal breast cancer cell line MCF10 [29]. First, G6PD and TKT were silenced in the breast cancer cell line MCF7 and the impact on cell proliferation, survival and cell cycle was assessed. Second, the metabolic effects of the inhibitions of these two enzymes were evaluated by [^13^C]-assisted metabolomics and metabolic flux analysis. Finally, the translational relevance of our findings was evaluated by survival and gene expression correlation analyses conducted on breast cancer patient datasets.

## RESULTS

### G6PD inhibition reduces cell proliferation and survival

**Figure 1:**
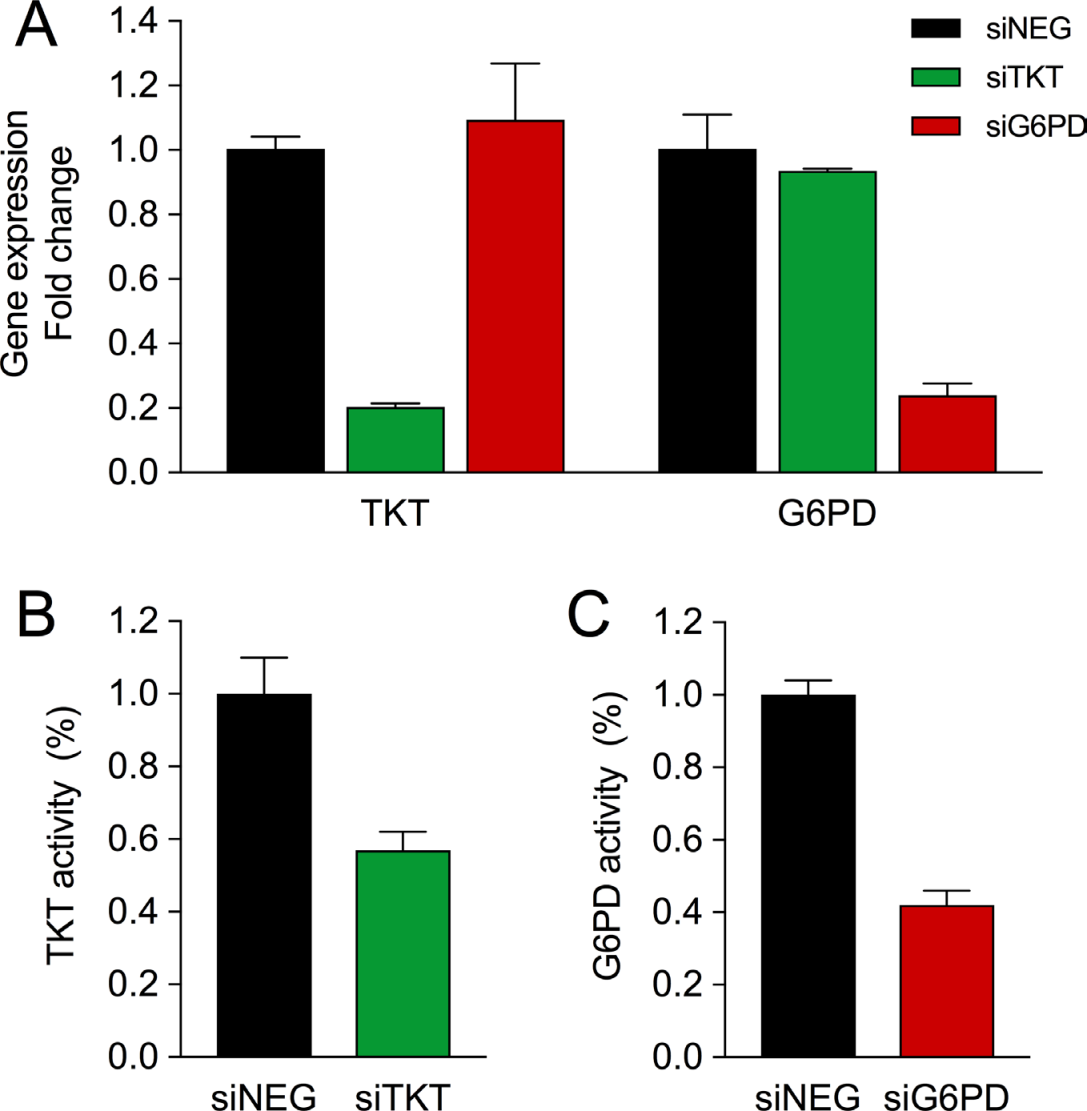
Silencing of TKT and G6PD in MCF7 cells (**A**) TKT and G6PD mRNA levels three days after transfection of non-targeting siRNA (siNEG) or siRNA targeting TKT (siTKT) or G6PD (siG6PD). (**B**) TKT activity five days after siTKT transfection. (**C**) G6PD activity after siG6PD transfection. In all plots values are expressed as fold change versus siNEG and bars represent mean (*n* = 3) ± SD.

**Figure 2:**
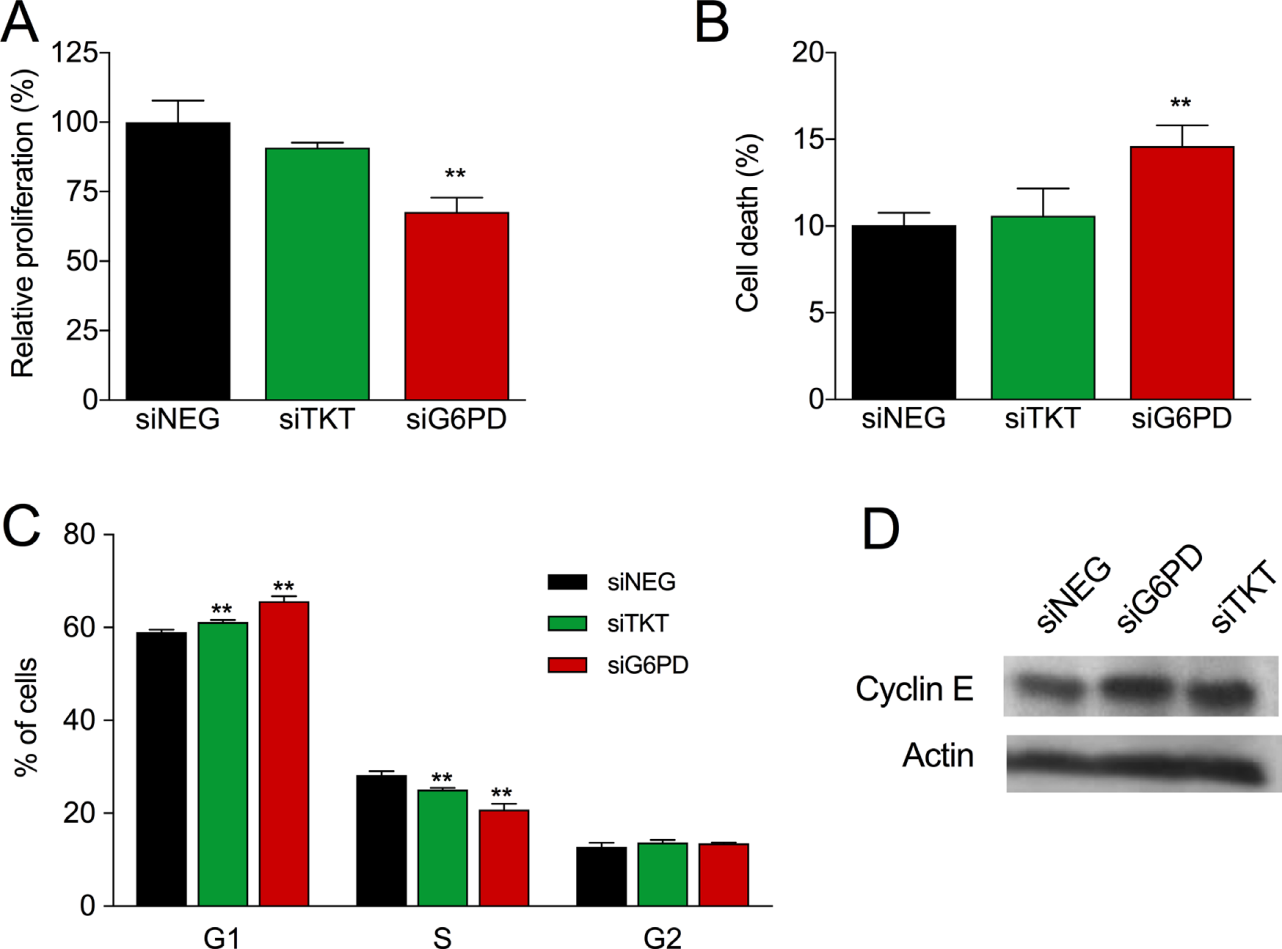
Role of PPP enzymes TKT and G6PD in cell proliferation, survival and cell cycle (**A**) Effect of TKT and G6PD silencing on proliferation six days after transfection expressed as percentage of siNEG. (**B**) Effect of TKT and G6PD silencing on cell death six days after transfection (percentage of PI-positive cells). Bars in A and B represent mean (*n* = 2) ± SEM for TKT and mean (*n* = 5) ± SEM for G6PD. (**C**) Effect of TKT and G6PD silencing on cell cycle progression five days after siRNA transfection. The percentages of cells in each phase are depicted as mean (*n* = 3) ± SD. (**D**) Cyclin E and β-actin (loading control) protein levels were analyzed four days after siRNA transfection.

To understand the biological and metabolic role of these two enzymes in breast cancer, we separately silenced TKT (siTKT) and G6PD (siG6PD) in the breast cancer cell line MCF7 by using specific small interference RNAs (siRNA). As shown in Figure 1A, both enzymes were effectively inhibited at the mRNA level by more than 75%, and the specific enzyme activities were reduced approximately 50% in both cases (Figure 1B–1C). Since PPP is an anabolic pathway that plays a fundamental role in cell growth, we examined the role of TKT and G6PD in proliferation and survival by performing proliferation experiments in combination with propidium iodide (PI) staining. G6PD silencing reduced cell proliferation (Figure 2A) and increased cell death as reported by an increase in the PI-positive cell population (Figure 2B). In contrast, no differences in proliferation and cell viability were detected when TKT was silenced (Figure 2A–2B). G6PD and TKT enzymatic activities have been reported to be enhanced during late G1 and S cell cycle phases and specific upregulation of the oxidative branch has been demonstrated in colon cancer cells [30]. Moreover, knowing that one of the essential roles of PPP is to provide ribose for cell proliferation, we hypothesized that inhibition of these enzymes could affect cell cycle progression. We found that both G6PD and TKT silencing induced a cell cycle arrest in G1 phase and a subsequent decrease in the percentage of cells in S phase (Figure 2C). This finding was also corroborated by an increase in the expression of cyclin E, a cell cycle-dependent protein that reaches its maximal expression in late G1 (Figure 2D).

### PPP inhibition modulates glycolytic flux and enhances glutaminolysis

**Figure 3:**
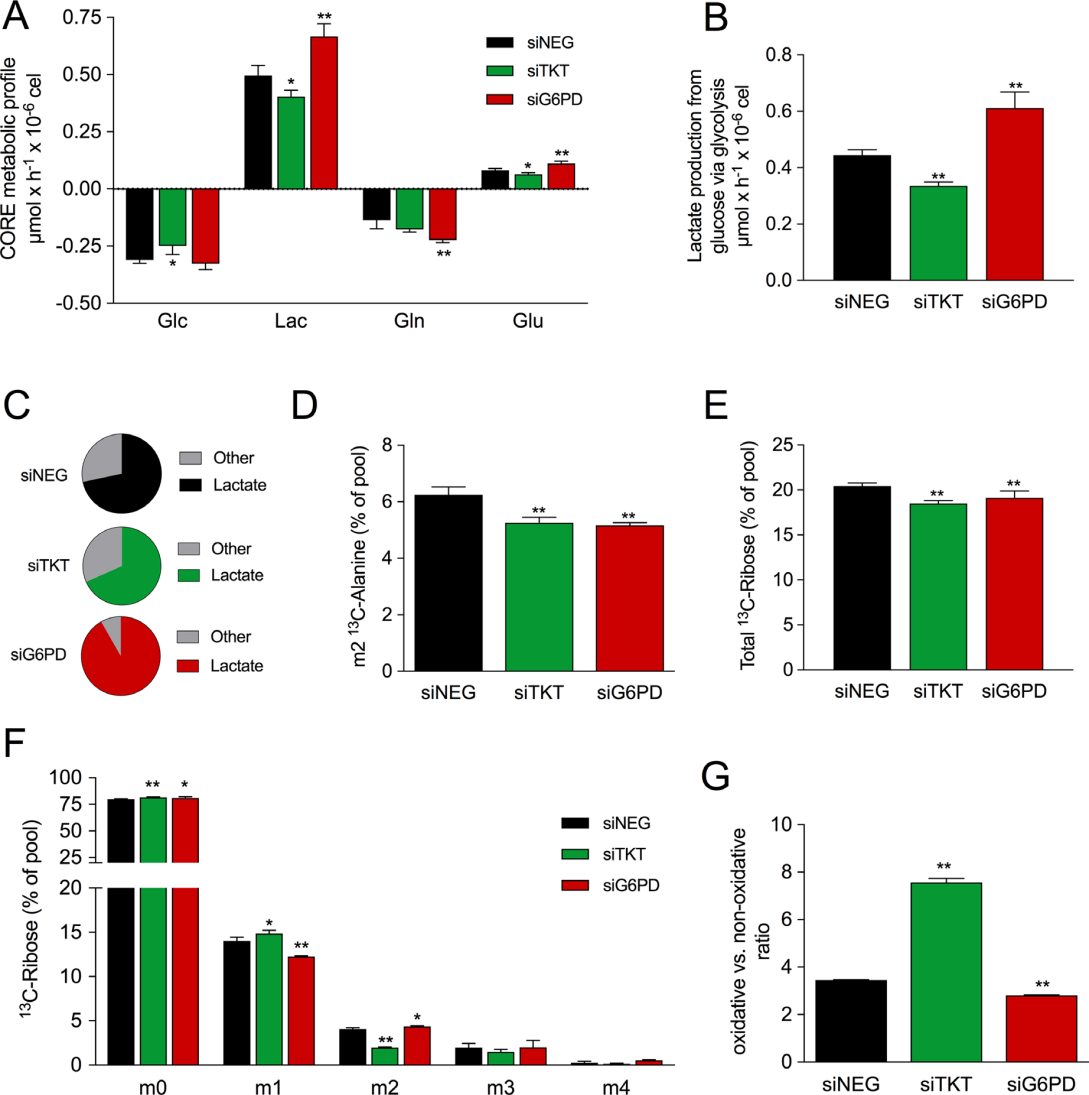
Metabolic effects of TKT and G6PD silencing [^13^C]-assisted metabolomics experiment was performed by replacing culture media by fresh media containing 50% of [1,2-^13^C_2_]-glucose four days after siRNA transfection. Cells were incubated with the tracer for 24h and extracellular fluxes and isotopologue distribution of different metabolites were determined. (**A**) CORE (consumption and release) metabolic profile showing rates of consumption of glucose (Glc) and glutamine (Gln) and release of lactate (Lac) and glutamate (Glu). (**B**) Flux of production of lactate from glucose via glycolysis. (**C**) Proportion of consumed glucose dedicated to either production of lactate or other uses. (**D**) Levels of m2 ^13^C-alanine in media. (**E**) Total ^13^C-labeled ribose from RNA. (**F**) Isotopologue distribution of ribose from RNA. (**G**) Ratio of m1 ^13^C-ribose to m2 ^13^C-ribose as an indicator of the oxidative vs. non-oxidative PPP activity. In all cases values represent mean (*n* = 3) ± SD.

To obtain further insight into the metabolic role of PPP in breast cancer cell metabolism, we performed a [^13^C]-assisted metabolomics experiment. Cells were transfected with the corresponding siRNAs and cultured in medium containing 50% of [1,2-^13^C_2_]-glucose. We first wanted to know whether the inhibition of G6PD and TKT altered the consumption and release (CORE) of the most abundant metabolites. TKT silencing decreased the rate of glucose consumption concomitantly with a decrease in the rate of lactate production (Figure 3A). On the contrary, silencing of G6PD did not affect glucose consumption, rather a significant increase in lactate production was observed. One of the most striking findings was that G6PD silencing doubled glutamine consumption and notably increased the production rate of glutamate. Conversely, when TKT was silenced no changes were observed in glutamine consumption but a decrease in the production of glutamate was detected.

Next, we sought to understand the mechanism for the increase in lactate production in G6PD-silenced cells. In cancer cells, lactate is primarily produced from glucose. When cells are cultured with [1,2-^13^C_2_]-glucose, the isotopologue distribution of lactate allows the estimation of the glucose-derived carbon flow. Using the rate of lactate production in combination with its isotopologue distribution (Supplementary Figure 1), the glycolytic flux—the rate of lactate production from glucose via glycolysis—can be estimated. Thus, when TKT was silenced, we observed a decrease in the glycolytic flux (Figure 3B). Strikingly, despite not observing changes in glucose consumption, silencing of G6PD promoted an increase in this flux. This fact prompted us to investigate the fate of glucose in the different experimental conditions. Silencing of G6PD raised the proportion of glucose-derived carbons dedicated to lactate, whereas this proportion did not change when TKT was silenced (Figure 3C). Also, it is noteworthy that silencing of TKT and G6PD reduced the percentage of m2 labelled alanine (Figure 3D).

### Silencing of TKT and G6PD reduces ribose synthesis

TKT and G6PD are the key enzymes of PPP and participate in the synthesis of ribose. Hence, we aimed to understand the impact of TKT and G6PD silencing on the PPP by analyzing the isotopologue distribution of ribose from RNA. Silencing of TKT and G6PD reduced in a similar extent the synthesis of ribose as indicated by the decrease of the [^13^C] enrichment of this metabolite (Figure 3E). The use of [1,2-^13^C_2_]-glucose allowed us to discriminate between the use of the oxidative and the nonoxidative branches of the PPP in the synthesis of ribose (ox-PPP and nonox-PPP, respectively). [1,2-^13^C_2_]-glucose can be metabolized through either the ox-PPP or the nonox-PPP branch, producing m1 or m2 ^13^C-labelled ribose, respectively. In our experiment, we observed that silencing of TKT caused a prominent decrease in the utilization of the nonox-PPP (m2 ribose) and slightly increased the flux through the ox-PPP (m1 ribose) (Figure 3F). On the contrary, silencing of G6PD reduced the ox-PPP flux and caused a marginal increase in the nonox-PPP flux. These changes altered the ox-PPP/nonox-PPP ratio, which is measured by the ratio of m1 to m2 ribose. Silencing of TKT dramatically increased the ox-PPP/nonox-PPP ratio whereas silencing of G6PD resulted in a modest reduction (Figure 3G).

### G6PD silencing increases ROS levels and reduces fatty acid synthesis

**Figure 4:**
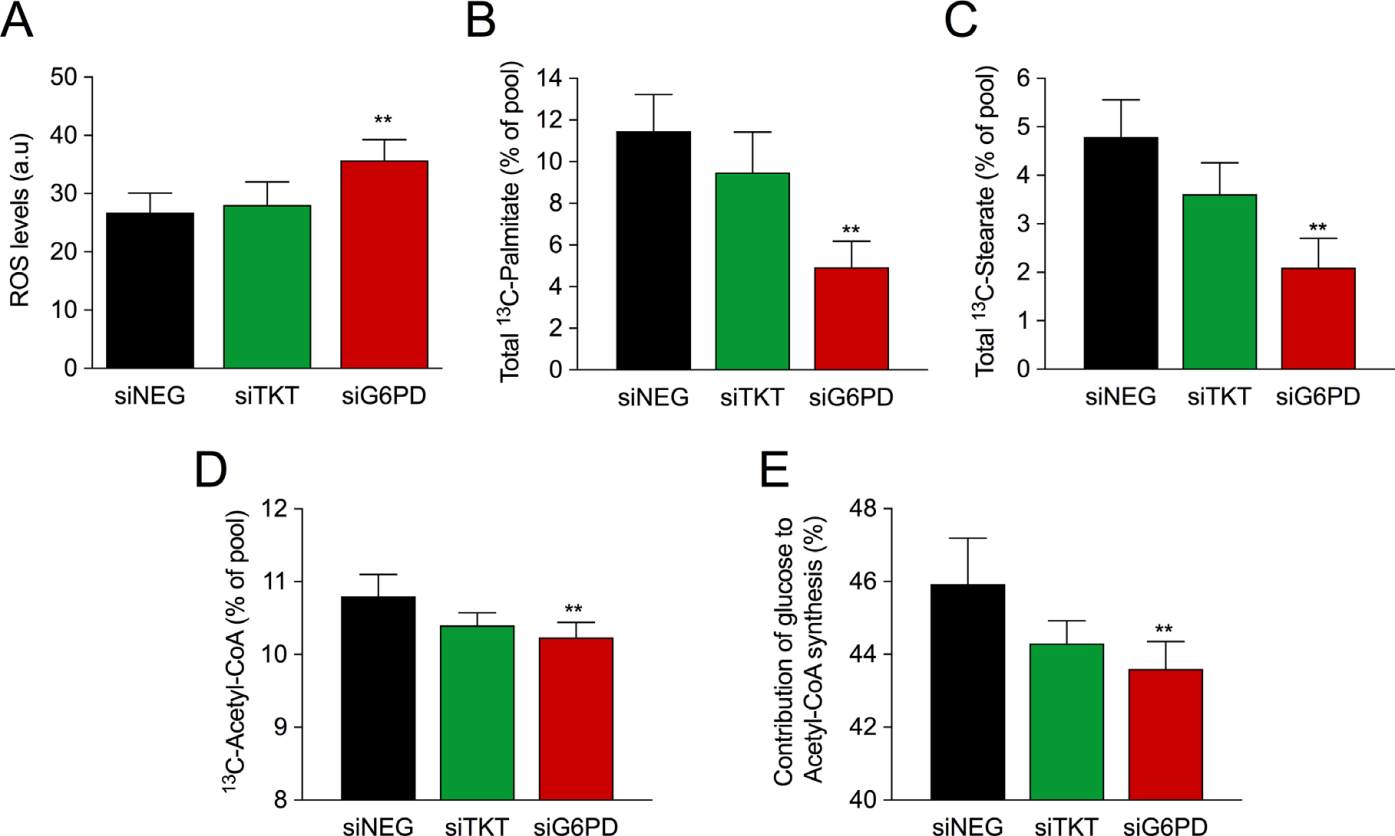
Role of PPP enzymes in ROS levels and lipid synthesis (**A**). ROS levels five days after transfection. Bars represent mean (*n* = 3) ± SEM. (**B**) Total ^13^C-labeled palmitate and (**C**) stearate. (**D**) Estimated fraction of ^13^C-acetyl-CoA depicted as percentage of the total pool. (**E**) Percentage of glucose dedicated to the synthesis of acetyl-CoA estimated using the fraction of ^13^C-acetyl-CoA in combination with the maximal theoretical enrichment of acetyl-CoA from glucose. Data shown in figures D and E was calculated as described in Boren et al 2003 [32]. Bars represent mean (*n* = 3) ± SD.

The ox-PPP is a major source of NADPH, an essential cofactor in ROS detoxification and lipid synthesis. As redox homeostasis plays an important role in breast cancer progression, we assessed the effect of TKT and G6PD silencing on ROS levels. Our results clearly showed that silencing of G6PD increased ROS levels whereas no changes were detected when TKT was silenced (Figure 4A).

It has also been documented that cancer cells display an increased requirement of lipids to sustain rapid proliferation [31]. Therefore, we studied the impact of TKT and G6PD silencing on lipid synthesis from glucose by analyzing the isotopologue distribution of palmitate and stearate. Cells were transfected and cultured in medium containing 50% of [1,2-^13^C_2_]-glucose. Remarkably, silencing of G6PD reduced the synthesis of palmitate and stearate as indicated by lower ^13^C enrichment in these fatty acids (Figure 4B–4C), whereas no changes in the enrichment of either of them were detected when TKT was silenced. We have previously demonstrated that silencing of G6PD increases the amount of glucose dedicated to lactate production. Therefore, the observed reduction in lipid synthesis was likely due to a decreased entry of pyruvate into mitochondria. To better understand this aspect, we next used the isotopologue distribution of palmitate to calculate the percentage of ^13^C-acetyl-CoA and the percentage of glucose dedicated to fatty acid synthesis. Thus, when G6PD was silenced, we observed a decrease in the percentage of ^13^C-acetyl-CoA, indicating a decreased flux of glucose-derived carbons into the mitochondria (Figure 4D). Consequently, we also found a reduction in the percentage of glucose dedicated to the synthesis of acetyl-CoA when G6PD was silenced (Figure 4E). Taken together, these results highlight the importance of the ox-PPP as a source of NADPH and the fine regulation that it exerts on redox balance and lipid synthesis.

### High expression of G6PD and TKT show positive correlation and is associated with poor patient outcome

**Figure 5:**
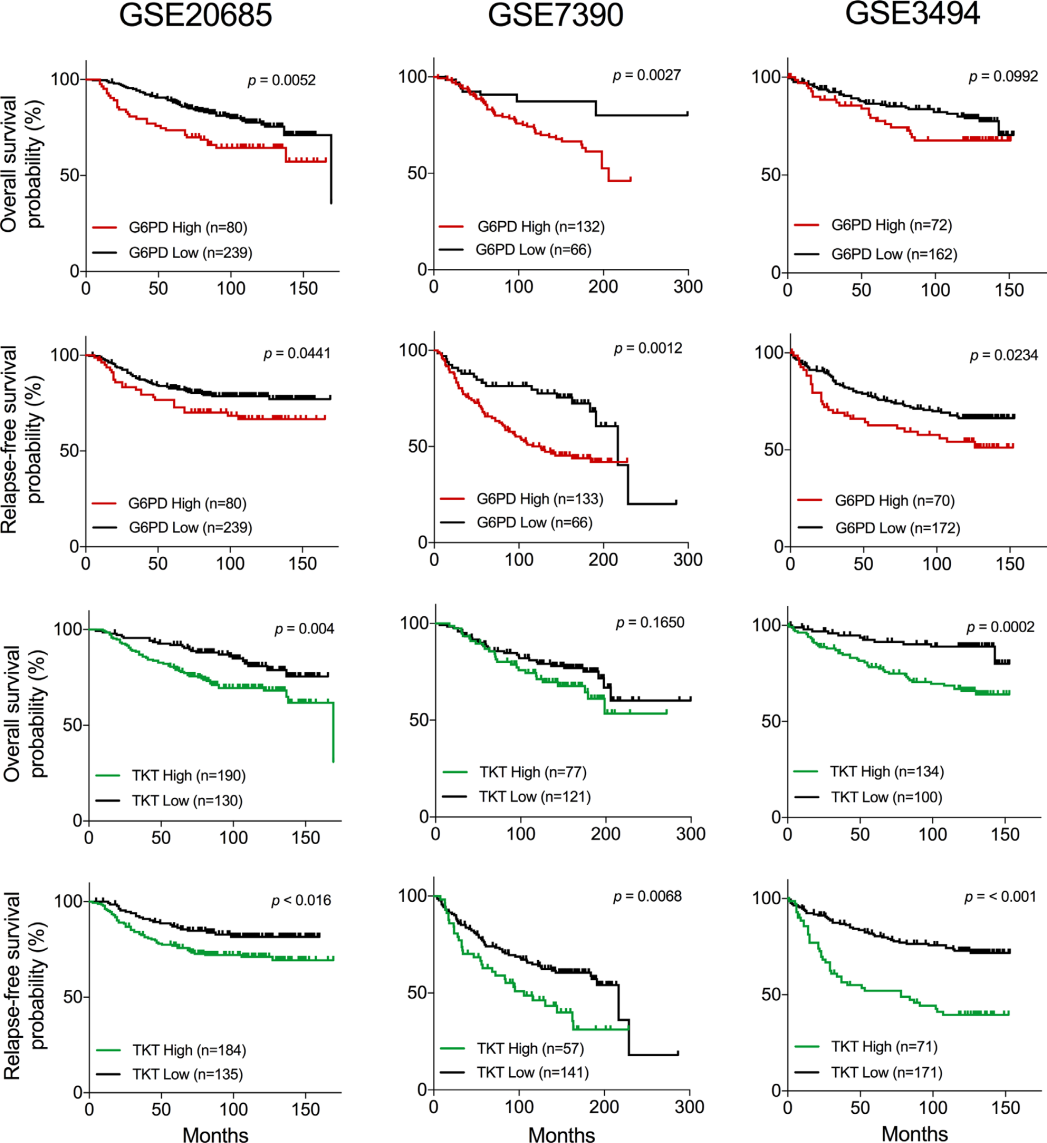
Survival analysis of breast cancer patients associated to the expression of TKT and G6PD Kaplan–Meier survival analysis showing relapse-free and overall survival for breast cancer patients with high and low expression levels of G6PD or TKT in three independent datasets. *p*-values of log-rank test are depicted in each plot.

**Figure 6:**
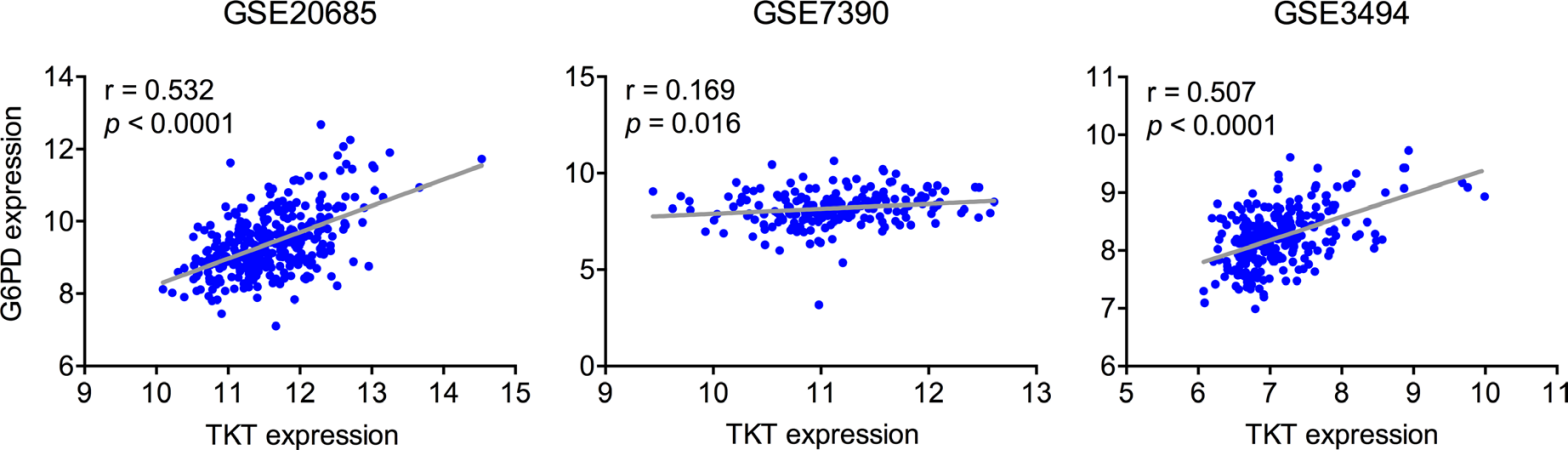
Gene expression correlation analyses Pearson’s correlation analysis showing gene expression levels of TKT and G6PD in breast cancer patients from three independent datasets. Pearson’s correlation coefficient (r) and *p*-values are shown for each analysis.

TKT and G6PD have been postulated as relevant enzymes in tumor cells by different studies, but little is known about the impact of the expression level of these enzymes on disease progression and survival. We surveyed three independent breast cancer gene expression datasets to explore the relation between TKT and G6PD expression and patient outcome. Kaplan–Meier analyses revealed a decreased overall and relapse-free survival in patients with high expression levels of TKT or G6PD in at least two out of the three datasets analyzed (Figure 5). Additionally, we were also interested in understanding how the expression of these two enzymes was coordinated. We performed a correlation analysis for the expression of TKT and G6PD in the three datasets. As shown in Figure 6, TKT and G6PD expression showed a strong positive correlation in two of the datasets (GSE20685 and GSE3494) and modest but significant correlation in the third one (GSE7390).

## DISCUSSION

The aim of this study was to provide a better understanding of the role of PPP enzymes TKT and G6PD in breast tumors. Herein, we have first shown that silencing of G6PD reduces cell proliferation, induces cell death and promotes cell cycle arrest, whereas silencing of TKT also induces cell cycle arrest but does not seem to affect any of the other processes. We next demonstrated that silencing of G6PD increases glycolytic flux, glutamine consumption and ROS levels, and decreases glucose-derived synthesis of acetyl-CoA, palmitate and stearate. On the contrary, TKT silencing reduces glycolytic flux and has no detectable consequences on any of the other processes. We finally provided strong evidence to support the fact that high expression of TKT and G6PD is associated with poor outcome in breast cancer patients.

G6PD has been previously reported to be important for tumor growth and for antioxidant defense [33–35], although the exact mechanism by which it is important in the proliferation of breast cancer cells is still unclear. The high dependency of the MCF7 cell line on G6PD is clearly supported by the fact that by only inhibiting 50% of G6PD enzymatic activity a 35% decrease in cell proliferation was observed. Despite the fact that G6PD is the key enzyme in the ox-PPP, we have demonstrated that the impact of G6PD silencing on ribose synthesis is low. However, G6PD silencing substantially increased ROS levels, suggesting that, in MCF7 cells, G6PD is crucial for redox homeostasis but might be dispensable for ribose synthesis. The metabolic reaction catalyzed by G6PD is one of the major sources of NADPH in the cell, which is used for balancing intracellular oxidative stress [36, 37]. It is widely accepted that most tumors deal with increased levels of ROS, leading to intracellular conditions of high oxidative stress [18, 38]. Collectively, it is then plausible that, in our study, cell death induced by G6PD silencing is due to the deregulation of ROS homeostasis. The reduced availability of NADPH could also explain the decrease observed in fatty acid synthesis, which may further contribute to the reduction in cell proliferation.

Although G6PD had the greatest impact on cell proliferation and ROS levels, it is worth noting that silencing of TKT had the highest impact on pathway-specific ribose synthesis. The level of total ^13^C-labelled ribose was similarly decreased in both TKT- and G6PD-silenced cells, but TKT showed a greater regulatory capacity over the nonox-PPP than G6PD did over the ox-PPP. This fact could be related to the protein level of each enzyme in the cell. It has been reported that G6PD usually functions at around 2% of its total maximum enzymatic rate [4]. This fact suggests that the concentration of the enzyme could be in excess and its inhibition would have a low impact on the synthesis of ribose. This is corroborated by the fact that 50% inhibition of G6PD enzymatic activity provoked only a slight decrease in m1 ribose. On the contrary, our results indicated that TKT probably works at its maximum enzymatic rate, since 50% inhibition of the enzyme decreased m2 ribose in the same proportion. Interestingly, these results showed that TKT inhibition also increased m1 ribose, which could be due either to the impossibility of reintroducing ribose synthesized by the ox-PPP into glycolysis or to a compensatory activation of the flux through the ox-PPP, resulting in accumulation of m1 ribose. Taken together, these findings suggest that although both oxidative and nonoxidative PPP pathways are tightly connected, the functions of maintenance of ROS homeostasis and synthesis of ribose may be partially decoupled.

In our study, we have also observed that TKT and G6PD silencing modified the glycolytic flux in an opposite manner. Silencing of G6PD did not change glucose consumption but rather notably increased lactate production from glucose via glycolysis, indicating a reduction in the flux of glucose-derived carbons into mitochondria and other biosynthetic pathways, as suggested by the decrease in the percentage of m2 alanine. This observation could be explained on the basis of HIF (Hypoxia Inducible Factor) activation. The increase in ROS levels caused by G6PD inhibition can stabilize and activate HIF-dependent signaling pathway [39, 40], which in turn upregulates glycolysis and diverts pyruvate away from mitochondria by inactivation of the pyruvate dehydrogenase complex [41, 42]. This mechanism could fully explain the increase in the percentage of glucose dedicated to lactate production, the decrease in the synthesis of glucose-derived acetyl-CoA and the reduction in the contribution of glucose to lipid synthesis.

One of the most striking findings in this study is the connection between PPP and glutaminolysis. Glutamine is a major carbon and nitrogen source that participates in bioenergetics (synthesis of ATP), defense against oxidative stress (provides carbons for glutathione synthesis and fuels NADPH-producing reactions) and production of macromolecules (lipids and other amino acids) [43]. Hence, the increase in the rate of glutamine uptake displayed by G6PD-silenced cells could be indicative of a response mechanism to provide carbons for NADPH-generating mitochondrial enzymatic reactions, such as those catalyzed by the malic enzyme and isocitrate dehydrogenase [43]. Likewise, it could also be a response mechanism aimed to maintain ATP levels. It has been described that MCF7 cells produce 80% of total ATP through mitochondrial oxidative phosphorylation [44]. Therefore, it seems plausible that the reduction of the pyruvate influx into the mitochondria resulted in increased utilization of other mitochondrial substrates, such as glutamine, for ATP synthesis [45]. Another potential explanation arises from our observation that silencing of G6PD increased the glycolytic flux, increasing the concentration of intracellular lactate and consequently acidifying the intracellular milieu. It has been recently reported that acidosis enhances glutamine uptake and glutaminolysis by a mechanism partially dependent on p53 that involves upregulation of GLS2 [46]. Under these conditions, glutamine is utilized to increase NADPH production and counteract the increase in ROS associated with acidosis.

Finally, we have also shown that high expression levels of G6PD and TKT correlate with poor overall and relapse-free survival in several breast cancer patient datasets. Previous studies in breast cancer tumors reported a gradual overactivation of PPP as the tumor progresses [23, 29]. Therefore, this fact alongside with our data suggests that the higher the expression of PPP enzymes, the higher the aggressiveness of the tumor. This also highlights not only the clinical relevance of this metabolic pathway, but also its potential use as a therapeutic target and biomarker of prognosis. In the same datasets, we have found that the expression of TKT and G6PD is positively correlated. PPP enzymes have been reported to be regulated by the transcription factor NRF2 [9]. NRF2 exerts pleiotropic effects in cancer cells. It participates in the regulation of the antioxidant defense, chemoresistance, tumorigenesis and tumor progression, in part by promoting anabolic reactions such as those catalyzed by TKT and G6PD. Inactivating mutations and overexpression of sequestrating proteins of the NRF2 negative regulator KEAP1 have been found in breast cancer, resulting in high protein levels of this transcription factor [47–49]. It is also noteworthy that PI3K-Akt pathway is frequently mutated in breast tumors [50] and other studies have reported that activation of this signaling pathway augments nuclear accumulation of NRF2 [9]. Collectively, all these observations suggest that the expression of TKT, G6PD and potentially the entire pathway could be coordinately regulated as part of a central metabolic reprogramming.

In conclusion, in this work we show that inhibition of PPP enzymes TKT and G6PD results in different metabolic phenotypes, and that overexpression of both enzymes correlate with poor patient prognosis. In particular, G6PD arises as an interesting candidate for therapeutic intervention, as it regulates key metabolic processes in tumor cells, such as synthesis of ribose, fatty acids and redox homeostasis. We believe that this study warrants further investigation to explore the potential of G6PD inhibition as a cancer treatment strategy and/or biomarker of prognosis.

## MATERIALS AND METHODS

### Cell culture

Breast cancer cell line MCF7 was purchased from ATCC and cultured following manufacturer’s instructions in MEM medium without phenol red (Gibco) containing 10% Fetal Bovine Serum (FBS) (Sigma-Aldrich), 10 mM glucose (Sigma-Aldrich), 1 mM pyruvate (Biological Industries), 2 mM glutamine (Gibco), 0.1% antibiotic (Penicillin 10 Units/mL-Streptomycin 10 µg/mL, Gibco), 0.01 mg/mL insulin (Sigma-Aldrich) and 1% non-essential amino acids (Biological Industries).

### siRNA transfection

MCF7 cells were transfected with siRNAs using Metafectene (Biontex) according to the manufacturer’s instructions. Briefly, 1 × 10^5^ cells/well were seeded in 6-well plates and after 24h were transfected with 60 nM of control siRNA (siNEG), siRNAs against TKT (siTKT) or G6PD (siG6PD). The siRNA sequences (Dharmacon) used were: siNEG, ON-TARGETplus Non-targeting siRNA D-001810-03-20 (sequence not provided by the manufacturer); siTKT, ON-TARGETplus J-004734-06-0010, GGAACUAGCCGCCAAUACA; siG6PD, ON-TARGETplus J-008181-06-0010, GAGAGUGGGUUUCCAGUAU.

### Proliferation, viability and cell cycle

Proliferation and viability assays were performed by flow cytometry combining cell counting and propidium iodide (PI) staining. At the end of the experiment or as indicated elsewhere cells were trypsinized and resuspended in 500 µL of a solution consisted of 450 µL of complete media, 45 µL of Flow-Count Fluorospheres (Beckman Coulter) and 5 µL of 1 mg/mL PI solution. Flow cytometer was set to 1 × 10^4^ fluorospheres cut-off and total cell number (PI-positive and PI-negative) was recorded. For cell cycle analysis, cells were collected and fixed with 70% cold ethanol prior to centrifugation and resuspension in PBS supplemented with 0.01% (v/v) Triton X-100, 1 mg/mL PI, and 0.2 mg/mL RNAse A (REAL Laboratories). Samples were analyzed by flow cytometry and cell cycle phase distribution was determined using FlowJo^®^.

### Western blotting

Protein extracts were prepared using RIPA buffer supplemented with 1% protease inhibitor cocktail (Thermo Fisher Scientific Inc.), 1% phosphatase inhibitor cocktail (Thermo Fisher Scientific Inc.) and quantified by BCA assay. Equal amounts of protein were separated by SDS-PAGE and transferred onto PVDF membrane (Bio-Rad Laboratories). After, membranes were blocked for 1h with 5% skim milk in 0.1% Tween PBS and incubated with the specific primary antibodies overnight at 4°C. Then, membranes were treated with the appropriate secondary antibody for 1 h at room temperature. All blots were treated with Immobilon ECL Western Blotting Detection Kit Reagent (EMD Millipore) and developed after exposure to an autoradiography film (VWR International). The following antibodies were used: anti-cyclin E (HE12, Santa Cruz) and anti-actin (691001, Millipore).

### RNA extraction and gene expression

RNA was extracted using Trizol^®^ (Invitrogen) following the manufacturer’s instructions. Briefly, cells were treated with Trizol and the homogenates were mixed with chloroform and centrifuged. RNA was then precipitated from the aqueous phase with cold isopropanol and subsequent centrifugation. Next, the supernatant was removed and the RNA pellet was rinsed with 75% cold ethanol**.** After ethanol evaporation, RNA was resuspended in RNAse-free water and the concentration was determined using Nanodrop. Next, cDNA was synthesized using 1 µg of RNA, random primers (Roche) and M-MLV reverse transcriptase (Invitrogen) according to the manufacturer’s indications. Gene expression analyses were performed by qPCR (ABI Prism 7700 Sequence Detector System, Applied Biosystems) using Taqman^®^ (Applied Biosystems) as per manufacturer’s instructions. The following probes were used: *G6PD,* Hs00166169_m1; *TKT,* Hs00169074_m1; *PPIA,* Hs99999904_m1. Gene expression was quantified by the ΔΔCt method using *PPIA* as a reference gene.

### ROS

Total intracellular ROS levels were determined by flow cytometry using H_2_DCFA probe (Invitrogen) following the manufacturer’s instructions. Briefly, cells were incubated with 5 µM H_2_DCFA in PBS for 30 min. After that, PBS was replaced by culture media and cells were incubated for 15 min at 37°C and 5% CO_2_, trypsinized and resuspended in a solution consisted of 50 µM H_2_DCFA and 20 µg/mL PI. Samples were analyzed by flow cytometry and only PI-negative cells were used for ROS quantification.

### Biochemical assays

The concentrations of glucose, lactate, glutamate and glutamine in culture medium were determined by spectrophotometry (COBAS Mira Plus, Horiba ABX) monitoring at 340 nm wavelength the production or consumption of NAD(P)H by specific enzymatic reactions for each metabolite. Glucose concentration was measured using the coupled enzymatic reactions of glucose-6-phosphate dehydrogenase and hexokinase (commercial enzymatic kit). The concentration of lactate was determined by the lactate dehydrogenase (LDH) reaction carried out at 37°C by adding a sample of media to a cuvette containing 1.55 mg/mL NAD^+^ and 87.7 U/mL of LDH in 0.2 M hydrazine 12 mM EDTA buffer, pH 9. Determination of glutamate concentration was performed through the glutamate dehydrogenase (GLDH) reaction at 37 °C by adding a sample of media to a cuvette containing 2.41 mM ADP, 3.9 mM NAD^+^ and 39 U/mL of GLDH in 0.5 M glycine/0.5 M hydrazine buffer, pH 9. The concentration of glutamine was determined by its conversion first to glutamate through the glutaminase (GLS) reaction followed by the quantification of glutamate concentration as described above. GLS reaction was carried out for 90 min at 37°C with shaking by adding a sample of culture media to a cuvette containing a mixture consisted of 90 mU/mL GLS in 111 mM acetate buffer, pH 5. To calculate the consumption/production rate of each metabolite, samples of culture media were taken at the beginning and at the end of the experiment and stored at –20°C for subsequent analysis. In the same cell culture plates, or parallel plates when required, cell number was determined through the procedure described above for normalization*.*

### [^13^C]-assisted metabolomics

Cells were grown in full medium containing 50% of [1,2-^13^C_2_]-glucose (Sigma-Aldrich) for 24 hours. At the end of the experiment, media and cell pellets were collected. Medium samples were kept at –20°C for ulterior determination of the concentration of glucose, lactate, glutamate and glutamine and the isotopologue distributions of glucose, lactate and alanine. Pellets were obtained by trypsinization and kept at –20°C for subsequent analysis of the isotopologue distribution of ribose and fatty acids. The isotopologue distribution analyses of the polar metabolites were performed by gas chromatography coupled to mass spectrometry (GC-MS) using an Agilent 7890A GC equipped with a HP5 capillary column coupled to an Agilent 5975C MS. GCMS-QP 2012 Shimadzu equipped with a bp × 70 (SGE) column was used for fatty acids analysis. In all cases, one microliter of sample was injected at 250°C using helium as carrier gas at a flow rate of 1 mL/min. Glucose was isolated from cell culture media using a tandem set of Dowex-1X8/Dowex-50WX8 ion-exchange columns using water as eluent. Samples were then dried under airflow and 2% (v/v) hydroxylamine hydrochloride in pyridine was added at 100°C for 30 min followed by addition of acetic anhydride for 1h. Samples were evaporated under N_2_ and dissolved in ethyl acetate. GC-MS analysis of glucose was performed under chemical ionization mode. Samples were injected at 250°C and oven temperature was held at 230°C for 2 min after injection and increased to 260°C at 10°C/min. Glucose retention time (RT) was 3.8 min. Detector was run in selected ion monitoring mode (SIM) and the ion abundance of the C1-C6 molecule (327–336 m/z) was recorded. Lactate was isolated from cell culture medium by acidification of samples with HCl, extraction with ethyl acetate and evaporation under N_2_. Next, dimethoxypropane and methanolic chloride were added for 1h at 75°C. After, samples were treated with n-propilamine for 1h at 100°C and dried under N_2_. The precipitate was then resuspended in dichloromethane and heptafluorobutyric anhydride at room temperature for 10 min. After that, samples were dried and resuspended in dichloromethane. GC-MS analysis of lactate was performed under chemical ionization mode. Sample was injected at 200°C and oven temperature was held at 100°C for 3 min after injection and increased to 160°C at 20°C/min. Lactate RT was 5.4 min. Detector was run in SIM and the ion abundance of the C1-C3 molecule (327–332 m/z) was recorded. Alanine was isolated from cell culture medium by passing a sample of media through a Dowex-50WX8 (H^+^) column and eluted with 2 N NH_4_OH followed by evaporation under airflow. Samples were then incubated in butanolic HCl at 100°C for 1 h and subsequently dried under N_2_. The precipitate was then dissolved in dichloromethane and trifluoroacetic anhydride and incubated at room temperature for 20 min. Samples were dried under N_2_ and dissolved in dichloromethane. GC-MS analysis of alanine was performed under chemical ionization mode. Sample was injected at 250°C and oven temperature was held at 110°C for 1 min, increased to 125°C at 10 °C/min, then to 153°C at 5°C/min, to 200°C at 50°C/min, to 216°C at 5°C/min and finally to 250°C at 25°C/min. Alanine RT was 5.28 min. Detector was run in SIM and the ion abundance of the C1-C3 molecule (m/z 241-246) was recorded. Ribose from RNA was isolated from cell pellets. After addition of Trizol^®^ (Invitrogen), the aqueous phase was separated and RNA was hydrolyzed by addition of 2N HCl for 2h at 100°C. Samples were then dried under airflow and derivatized as previously described for glucose. GC-MS analysis of ribose was performed under chemical ionization mode. Sample was injected at 250°C and oven temperature was held at 150°C for 1 min after injection, increased to 275°C at 15°C/min and finally to 300°C at 40°C/min. Ribose RT was 5.3 min. Detector was run in SIM and the ion abundance of the C1-C5 molecule (256–261 m/z) was recorded. Palmitate and stearate from cultured cells were extracted from the inter- and lower phase of the Trizol extract as described above by adding 100% ethanol and 30% KOH. After that, samples were incubated at 70°C overnight. Then, free fatty acids were extracted by addition of petroleum ether and samples were dried under N_2_. Fatty acids were then derivatized by adding 0.5 N methanolic-HCl at 70°C for 1h. GC-MS analysis of palmitate and stearate was performed under chemical ionization mode. Sample was injected at 250°C and oven temperature was held at 120°C for 1 min after injection and increased to 220°C at 5°C/min. Detector was run in SIM and the ion abundance in the range of 269-278 m/z for palmitate (RT: 9.2) and 297–307 m/z for stearate (RT:11.85) was recorded.

### Enzyme activities

Cells extracts were prepared using lysis buffer consisting of 20 mM Tris-HCl buffer, pH 7.5 supplemented with 1 mM DTT, 1 mM EDTA, 0.02% (v/v) Triton X-100, 0.02% (v/v) sodium deoxycholate and protease inhibition cocktail (Sigma-Aldrich). Lysates were then disrupted by sonication using titanium probe (VibraCell, Sonics & Materials Inc.) and immediately centrifuged at 12,000 × g for 20 min at 4°C. The supernatant was used for the determination of the enzyme activities by monitoring changes in the concentration of NAD(P)H using a COBAS Mira Plus analyzer. Glucose-6-phosphate dehydrogenase activity (G6PD, EC 1.1.1.49.) was measured by adding the lysates to a cuvette containing 0.5 mM NADP^+^ in 50 mM Tris-HCl buffer, pH 7.6, at 37°C. The reaction was initiated by the addition of glucose-6-phosphate up to a final concentration of 2 mM. Transketolase activity (TKT, EC 2.2.1.1.) was determined by adding the lysates to a cuvette containing 5 mM MgCl_2_, 0.2 U/mL of triose phosphate isomerase, 0.2 mM NADH and 0.1 mM thiamine pyrophosphate in 50 mM Tris-HCl buffer, pH 7.6, at 37°C. The reaction was initiated by adding the resulting solution of the reaction of 50 mM ribose-5-phosphate in 50 mM Tris-HCl buffer, pH 7.6, with 0.1 U/mL of ribulose-5-phosphate-3-epimerase and 1.7 mU/mL of phosphoriboisomerase for 1h in agitation at 37°C.

### Survival and correlation analysis

The Kaplan–Meier (KM) estimator was used to construct the survival curves. Survival analyses of the breast cancer datasets GSE20685 [51], GSE7390 [52], GSE3494 [53] were performed through the online application KM plotter [54] and subsequently edited in GraphPad PRISM. Time to death and time to relapse were chosen as the events of interest to estimate the overall (OS) and relapse-free survival (RFS). In each cohort patients were separated into high and low expression of TKT and G6PD as reported by the JetSet probes [55] 208700_s_at (TKT) and 202275_at (G6PD) by applying the best cutoff algorithm, which computes the KM estimator for each percentile of expression between the lower and upper quartiles and uses the best performing threshold as the final cutoff [56]. Statistical differences between survival curves were assessed by log-rank test and the corresponding *p*-values are indicated in each plot. Pearson’s correlation analyses were performed in PRISM using normalized TKT and G6PD expression values obtained from each dataset using the above-mentioned probe codes.

### Statistical analyses

Results are expressed as mean **±** SEM for multiple experiments or mean **±** SD for individual experiments. Data was analyzed by one-way ANOVA followed by Dunetts’s pos*t*-test analysis for correction of multiple comparisons against the control condition (siNEG). GraphPad PRISM was used for generation of plots and statistical tests. ^*^ denotes adjusted *p*-value < 0.1 and ^**^ *p*-value < 0.05.

## SUPPLEMENTARY MATERIALS FIGURE


