# Supplementary material for: Glucose-6-phosphate dehydrogenase and transketolase modulate breast cancer cell metabolic reprogramming and correlate with poor patient outcome
Source: Oncotarget. 2017 Oct 7;8(63):106693–706. doi: 10.18632/oncotarget.21601 (PMC5739767; doi:10.18632/oncotarget.21601)
Supplement: Supplementary file 1 [file oncotarget-08-106693-s001.pdf]

Glucose-6-phosphate dehydrogenase and transketolase modulate breast cancer cell metabolic reprogramming and correlate with poor patient outcome

SUPPLEMENTARY MATERIALS

GC-MS data reduction

Mass spectrometry data correction was performed by using regression analysis employing in-house developed software [1] as described in Aguilar et al. 2015 [2].

Calculation of the glycolytic flux

The flux of lactate produced from glucose via glycolysis can be estimated by combining the concentration and the isotopologue distribution of lactate exported to the media by cells cultured in the presence of 50% [1,2-$^{13}C_2$]-glucose. In order to calculate this flux, we assumed that MCF7 cells do not consume lactate in our experimental setting, so this metabolite is only produced. FBS contains unlabeled lactate that remains in the media during all the incubation time and contributes to the m0 of the total pool of lactate at the end of the incubation (t = 24). Taking this into account, we first estimated the isotopologue distribution of the lactate produced during the experiment. First, the percentage of each isotopologue of total lactate (Lac_{Tot}(m0,m1,m2,m3)$_{t=24}$ was multiplied by the final concentration of lactate $[Lac]_{t=24}$ to obtain the concentration of each isotopologue $[Lac_{Tot}(m0,m1,m2,m3)]_{t=24}$ at the end of the incubation period (t = 24) (Eq. 1):

$$[Lac_{Tot}(m0,m1,m2,m3)]_{t=24}\,(mM) = Lac_{Tot}(m0,m1,m2,m3)_{t=24}\,(\%) \times [Lac]_{t=24}\,(mM) \quad (1)$$

Next, the concentration of lactate at t = 0 $[Lac]_{t=0}$ was subtracted to the concentration of total m0 lactate at t = 24 $[Lac_{Tot}(m0)]_{t=24}$ to obtain the concentration of unlabeled produced lactate released during the experiment $[Lac_{Prod}(m0)]$ (Eq. 2). The concentrations of produced m1, m2 and m3 lactate $[Lac_{Prod}(m1,m2,m3)]$ coincide with the calculated concentrations of m1, m2 and m3 of total lactate $[Lac_{Tot}(m1,m2,m3)]_{t=24}$.

$$[Lac_{Prod}(m0)]\,(mM) = [Lac_{Tot}(m0)]_{t=24}\,(mM) - [Lac]_{t=0} \quad (2)$$

Next, the percentage of each isotopologue of produced lactate Lac_{Prod}(m0,m1,m2,m3) was then recalculated by dividing the concentration of each isotopologue of produced lactate $[Lac_{Prod}(m0,m1,m2,m3)]_{t=24}$ by the concentration of produced lactate $[Lac_{Prod}]$ (Eq. 3). The concentration of lactate produced during the experiment was estimated by subtracting the concentration of lactate at t = 0 $[Lac_{Tot}]_{t=0}$ to the concentration of lactate at t = 24 $[Lac_{Tot}]_{t=24}$ (Eq. 4).

$$Lac_{Prod}(m0,m1,m2,m3)_{t=24}\,(\%) = [Lac_{Prod}(m0,m1,m2,m3)]_{t=24}\,(mM) / [Lac_{Prod}]\,(mM) \quad (3)$$

$$[Lac_{Prod}]\,(mM) = [Lac_{Tot}]_{t=24}\,(mM) - [Lac_{Tot}]_{t=0}\,(mM) \quad (4)$$

Following, the glycolytic tax (GT), defined as the percentage of lactate produced from glucose through glycolysis, was calculated as described in Alcarraz-Vizan et al. 2010 [3]:

$$GT = Lac_{Prod}(m2)_{t=24}\,(\%) \times 2 / Glc(m2)_{t=0}\,(\%) \quad (5)$$

where $Glc(m2)_{t=0}$ is the percentage of [1,2-$^{13}C_2$]-glucose in the cell culture media at the beginning of the experiment (t = 0). Next, the maximum amount of lactate produced from glucose through glycolysis $[Lac_{ProdGlyc}]$ was obtained by multiplying the GT by the concentration of produced lactate $[Lac_{Prod}]$ (Eq. 6).

$$[Lac_{ProdGlyc}]\,(mM) = GT \times [Lac_{Prod}]\,(mM) \quad (6)$$

The concentration of lactate produced from glucose through glycolysis $[Lac_{ProdGlyc}]$ was then normalized by time and cell number.

REFERENCES

1. Selivanov VA, Benito A, Miranda A, Aguilar E, Polat IH, Centelles JJ, Jayaraman A, Lee PW-N, Marín S, Cascante M. MIDcor, an R-program for deciphering mass interferences in mass spectra of metabolites enriched in stable isotopes. BMC Bioinformatics. 2017; 18:88. https://doi.org/10.1186/s12859-017-1513-3.
2. Aguilar E, Marin de Mas I, Zodda E, Marín S, Morrish F, Selivanov V, Meca-Cortés Ó, Delowar H, Pons M, Izquierdo I, Celià-Terrassa T, de Atauri P, Centelles JJ, et al. Metabolic Reprogramming and Dependencies Associated with Epithelial Cancer Stem Cells Independent of the Epithelial-Mesenchymal Transition Program. Stem Cells. 2016; 34:1163–76. https://doi.org/10.1002/stem.2286.
3. Alcarraz-Vizán G, Boren J, Lee WNP, Cascante M. Histone deacetylase inhibition results in a common metabolic profile associated with HT29 differentiation. Metabolomics. 2010; 6:229–37. https://doi.org/10.1007/s11306-009-0192-0.

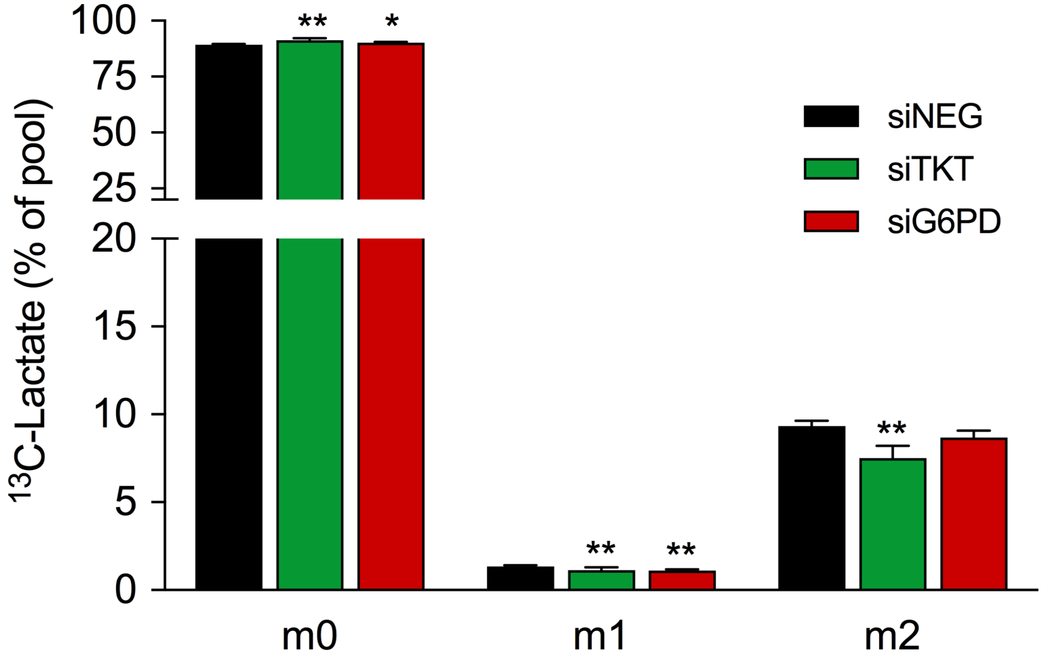

Supplementary Figure 1: Metabolic effects of TKT and G6PD silencing on lactate. Isotopologue distribution of lactate from media. Data was obtained under the same conditions as in Figure 3. Bars represent mean (n = 3) ± SD.
